# A Site-Specific Recombinase-Based Method to Produce Antibiotic Selectable Marker Free Transgenic Cattle

**DOI:** 10.1371/journal.pone.0062457

**Published:** 2013-05-01

**Authors:** Yuan Yu, Yongsheng Wang, Qi Tong, Xu Liu, Feng Su, Fusheng Quan, Zekun Guo, Yong Zhang

**Affiliations:** Key Laboratory of Animal Biotechnology of the Ministry of Agriculture, College of Veterinary Medicine, Northwest A&F University, Yangling, Shaanxi, People’s Republic of China; University of Connecticut, United States of America

## Abstract

Antibiotic selectable marker genes have been widely used to generate transgenic animals. Once transgenic animals have been obtained, the selectable marker is no longer necessary but raises public concerns regarding biological safety. The aim of this study was to prepare competent antibiotic selectable marker free transgenic cells for somatic cell nuclear transfer (SCNT). PhiC31 intergrase was used to insert a transgene cassette into a “safe harbor” in the bovine genome. Then, Cre recombinase was employed to excise the selectable marker under the monitoring of a fluorescent double reporter. By visually tracking the phenotypic switch from red to green fluorescence, antibiotic selectable marker free cells were easily detected and sorted by fluorescence-activated cell sorting. For safety, we used phiC31 mRNA and cell-permeant Cre protein in this study. When used as donor nuclei for SCNT, these safe harbor integrated marker-free transgenic cells supported a similar developmental competence of SCNT embryos compared with that of non-transgenic cells. After embryo transfer, antibiotic selectable marker free transgenic cattle were generated and anti-bacterial recombinant human β-defensin-3 in milk was detected during their lactation period. Thus, this approach offers a rapid and safe alternative to produce antibiotic selectable marker free transgenic farm animals, thereby making it a valuable tool to promote the healthy development and welfare of transgenic farm animals.

## Introduction

Transgenic farm animals are important materials for biomedical and agricultural research [1], [2]. However, the present approaches to generate transgenic animals are still hampered by low efficiency [3], variable expression levels of the transgene [4], and the residual antibiotic-resistance gene that is required to select transgenic cells but provokes public concerns regarding biological safety. Thus, efficient and safe methods are urgently needed to improve the current situation.

Currently, somatic cell nuclear transfer (SCNT) has been proven to be the most effective protocol for the production of transgenic animals [5], [6]. Therefore, preparation of competent transgenic donor cells is a key step for successful SCNT. Many methods are available to produce transgenic donor cells, and the traditional method relies on random integration of the transgene of interest. However, random integration into chromosomes suffers from low stable integration [7], and variable expression levels of the genes due to positional effects and the number of inserted copies [8]–[11]. Homologous recombination provides site specificity, but at a very low efficiency [12]. Furthermore, many virus-based gene transfer approaches are limited by their preference for integration into the gene-coding region [13], [14], which is a safety risk of transgenic animal production. Thus, an efficient and safe gene delivery approach is important for transgenic cell preparation.

PhiC31 integrase, the *Streptomyces* phage-derived recombinase, has been developed as a non-viral gene therapy tool, because it has the ability to integrate a transgene-containing plasmid carrying an *attB* site into pseudo *attP* sites in mammalian genomes [15], [16]. This enzyme has been previously shown to integrate genes effectively and prolong transgene expression in several mammalian cell culture systems including those for human keratinocytes [17], muscle-derived stem cells and myoblasts [18], and a human T cell line [19]. In addition, it has been recently reported that phiC31-mediated integration events usually occur in “genomic safe harbors” in mammalian cells [20], the regions of the genome where the integrated material is adequately expressed without perturbing endogenous gene structure or function, following a process that is amenable to precise mapping and minimizing occult genotoxicity [21], which makes phiC31 integrase an ideal tool for gene delivery and transgenic animal production.

Furthermore, the integration of antibiotic-resistance genes into transgenic animals may cause many problems such as disturbing the expression of neighboring genes [22], confounding the evaluation of food safety of these transgenic animals, and increasing worldwide public concern regarding the release of such antibiotics resistance genes into the environment. Recent studies have shown that selective marker genes can be successfully knocked out from transgenic cells using the Cre/loxP system [23]–[26]. However, because of the reversibility of Cre recombinase, labor-intensive procedures must be performed to identify complete excision events among randomly picked colonies. Although a lot of effort is being invested into solving this issue, an efficient and reliable method has yet to be developed.

Here, we demonstrate an efficient and safe approach to produce transgenic cattle, which consists of single-copy integration of *human β-defensin-3* (*HBD3*) gene into a genomic safe harbor and visual removal of the antibiotic-resistance marker. In addition, this procedure can be prospectively applied for breeding other antibiotic marker-free disease-resistant transgenic animals as well as production of human recombinant pharmaceuticals in transgenic cattle.

## Results

### Generation of Transgenic Cells using phiC31 Integrase mRNA

**Figure 1:**
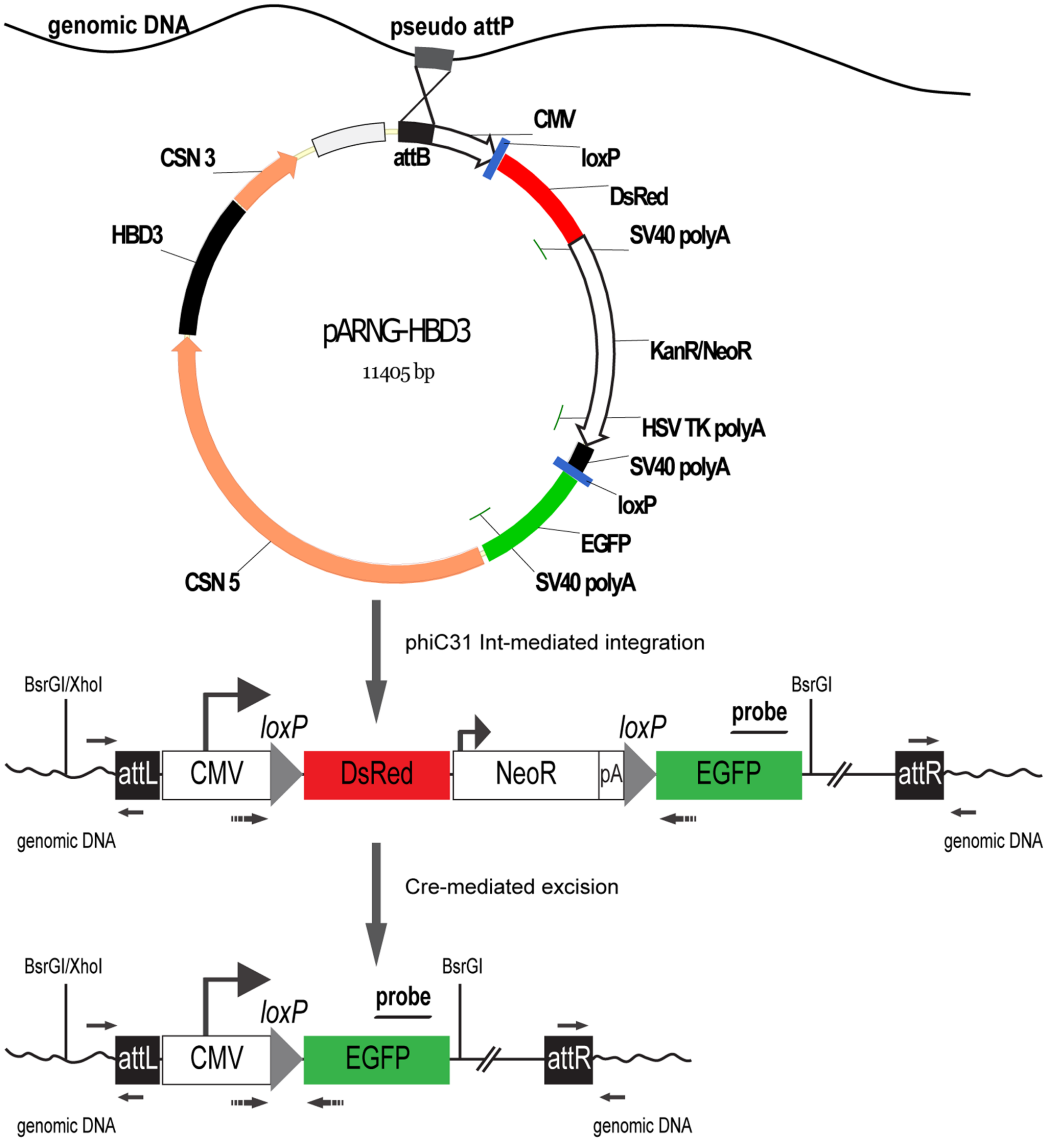
Schematic overview of the fluorescence double reporter construct pARNG-HBD3. The *attB* site is used for site-specific integration mediated by phiC31 integrase. *DsRed* is ubiquitously expressed under the control of the CMV promoter. A neomycin resistance gene (*neoR*) under the control of the SV40 early promoter allows selection of stable-transfected colonies. Cre-mediated recombination causes excision of *DsRed* and *neoR* resulting in expression of the second reporter, *EGFP*.

Delivery of the *human β-defensin-3* transgene into bovine fetal fibroblasts was performed by co-electroporation of phiC31 integrase mRNA produced by *in vitro* transcription, and a transgenic plasmid, pARNG-HBD3. The pARNG-HBD3 plasmid was an *attB*-containing human β-defensin-3 mammary gland expression vector (Fig. 1). A fluorescence double reporter was constructed to monitor Cre-mediated recombination in living cells before and after Cre recombination by expression of two different fluorescent proteins. The fluorescence double reporter construct contained the ubiquitous active CMV IE promoter that drove transcription of the fluorescent gene. Downstream of the CMV IE promoter was a *loxP*-flanked *DsRed* gene and P_SV40e_-driven neomycin-resistance (*neoR*) expression cassette coupled to an *enhanced green fluorescent protein* (*EGFP*) reporter.

To test the effect of phiC31 integrase on site-specific integration, 5 µg pARNG-HBD3 was electroporated into 1 × 10^6^ bovine fetal fibroblasts in the presence of 1 µg phiC31 integrase mRNA (integrase group) or 1 µg inactive mutant phiC31 integrase mRNA (mutant integrase group). At 8–12 days after electroporation, individual cell colonies were obtained by G418 screening. A total of 46 colonies were obtained by integrase mediated electroporation while 30 colonies were obtained by the mutant integrase mediated electroporation. Both G418-resistant and red fluorescent protein (RFP)-positive (RFP+) colonies were picked and expanded in culture. A total of 38 out of 46 G418-resistant colonies were RFP+ in the integrase group, whereas 18 out of 30 colonies were RFP+ in the mutant integrase group. These RFP+ colonies were further analyzed by PCR. A band of 426 bp for an un-cleaved *attB* site was found in 15 RFP+ colonies of the integrase group (Fig. S1a) and all 18 RFP+ colonies of the mutant integrase group (Fig. S1b), indicating that these colonies were involved with random integration events. The remaining 23 RFP+ colonies of the integrase group, which lacked the 426 bp band, were preliminarily considered to be the result of *attB* site cleavage caused by site-specific integration. These *attB*-cleaved RFP+ colonies were evaluated for the number of integration events by absolute quantity PCR. Among the 23 integrants, 11 exhibited single-copy integration, 12 exhibited double integration, and three or more integrants were not found in this study (Fig. S1c).

Of the 11 single-copy site-specific integrants we evaluated by half-nested inverse PCR, seven pseudo *attP* sites were identified. Among the seven sites, three sites were found in intergenic regions, the other four were located within an intron, and none of the sites were located in an exon. Notably, of the 11 single-copy integrants, five were found to be integrated in the intergenic regions on chromosome 2, whereas the others were identified only once as shown in Table 1. We found that repetitive sequences were expanded in DNA sequences surrounding these pseudo *attP* sites. Short interspersed elements (SINEs) were major repetitive elements flanking all integration sites, which included both intergenic and intronic insertions. The mammalian-wide interspersed repeat (MIR) and bovine tRNA pseudo gene coupled to A element (BovA) subfamilies were the most frequently identified types of SINEs. We also evaluated the integration sites obtained in our study according to the criteria articulated in a recently published study by Papapetrou et al. [21], which defined so-called “genomic safe harbors”. Of the seven integration sites, only the intergenic integration site on chromosome 2 met the criteria proposed by the previous study (Table S1). Using junction PCR with primers specific for the phiC31 integrase *attB* site and the genomic site, 13 out of 46 colonies of the integrase group were found to be integrated in the genomic safe harbor on chromosome 2, and demonstrated a total integration frequency of 28% (Fig. S1d). However, none of the 18 colonies of the mutant integrase group was detected to be integrated in the safe harbor on chromosome 2 (data not shown).

**Table 1:** Overview of the seven integration events analyzed by half-nested inverse PCR and sequencing.

Pseudo site	GenBankaccession No.	Chromosome	Within Intron	Intergenic		Repetitiveelements			Identity with WT *attP*
				Distance to upstream gene	Distance to downstreamgene	SINEs	DNA elements	Total length of repeats	
BFF2	NW_003103850	2		121 kb to IRS-1	1068 kb to KIAA1486	SINE/MIR,SINE/BovA		341 bp (63%)*	33%
BFF4a	NW_001494941	4	PODXL					–	39%
BFF4b	NW_003103903	4		3.4 kb to NAA38	1909 kb to KCND2	SINE/MIR		111 bp (21%)	33%
BFF13	NW_001493120	13	KIF5B				low complexity	30 bp (6%)	33%
BFF19	NW_003104490	19		5.9 kb to UNC45B	86 kb to SLFN11	SINE/MIR		111 bp (21%)	28%
BFF22	NW_003104541	22	LOC100852122			SINE/tRNA-Glu,SINE/MIR		256 bp (47%)	41%
BFF25	NW_001494275	25	CLCN7			SINE/BovA		33 bp (6%)	21%

* Total length of interspersed repeats/540 bp of genomic sequences flanking phiC31 integration sites.

### Excision of the Antibiotic Selectable Marker from Transgenic Cells using Cell-permeant Cre Recombinase

**Figure 2:**
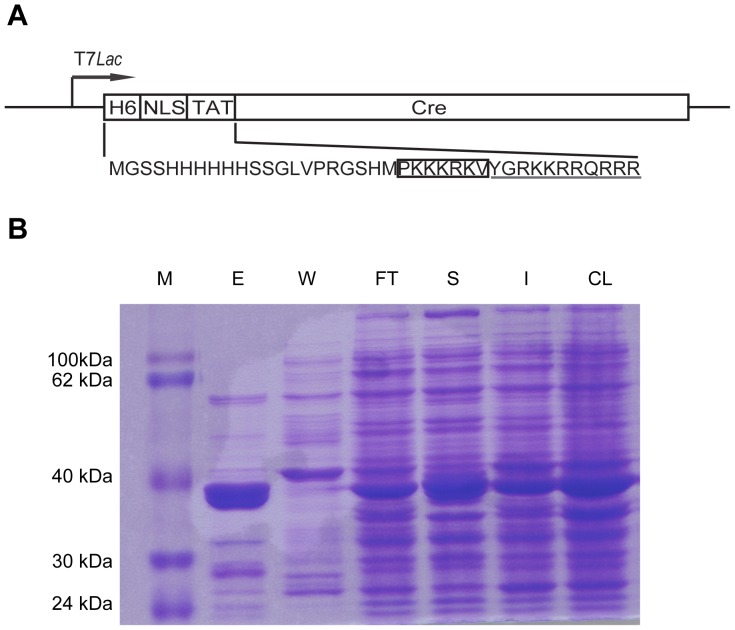
Recombinant modified Cre protein-mediated recombination. (a) Schematic of cell-permeant His-NLS-TAT-Cre fusion protein. H6, 6×His tag. The amino acid sequence of NLS is boxed and TAT is underlined. (b) Purification of recombinant His-NLS-TAT-Cre protein from bacteria as analyzed by Coomassie blue staining of an SDS-PAGE gel. CL: Cleared lysate; I: Insoluble; S: Supernatant; FT: Flow-through; W: Washing; E: Eluate; M: Marker.

Cell-permeant Cre protein was used to remove the selectable *neoR* marker gene. His-NLS-TAT-Cre protein (Fig. 2a) used in this study is a recombinant fusion protein consisting of a basic protein translocation peptide derived from HIV-TAT (TAT), a nuclear localization sequence (NLS) derived from SV40 large T antigen, the Cre protein and an N-terminal histidine tag for efficient purification from *E. coli* (Fig. 2b).

**Figure 3:**
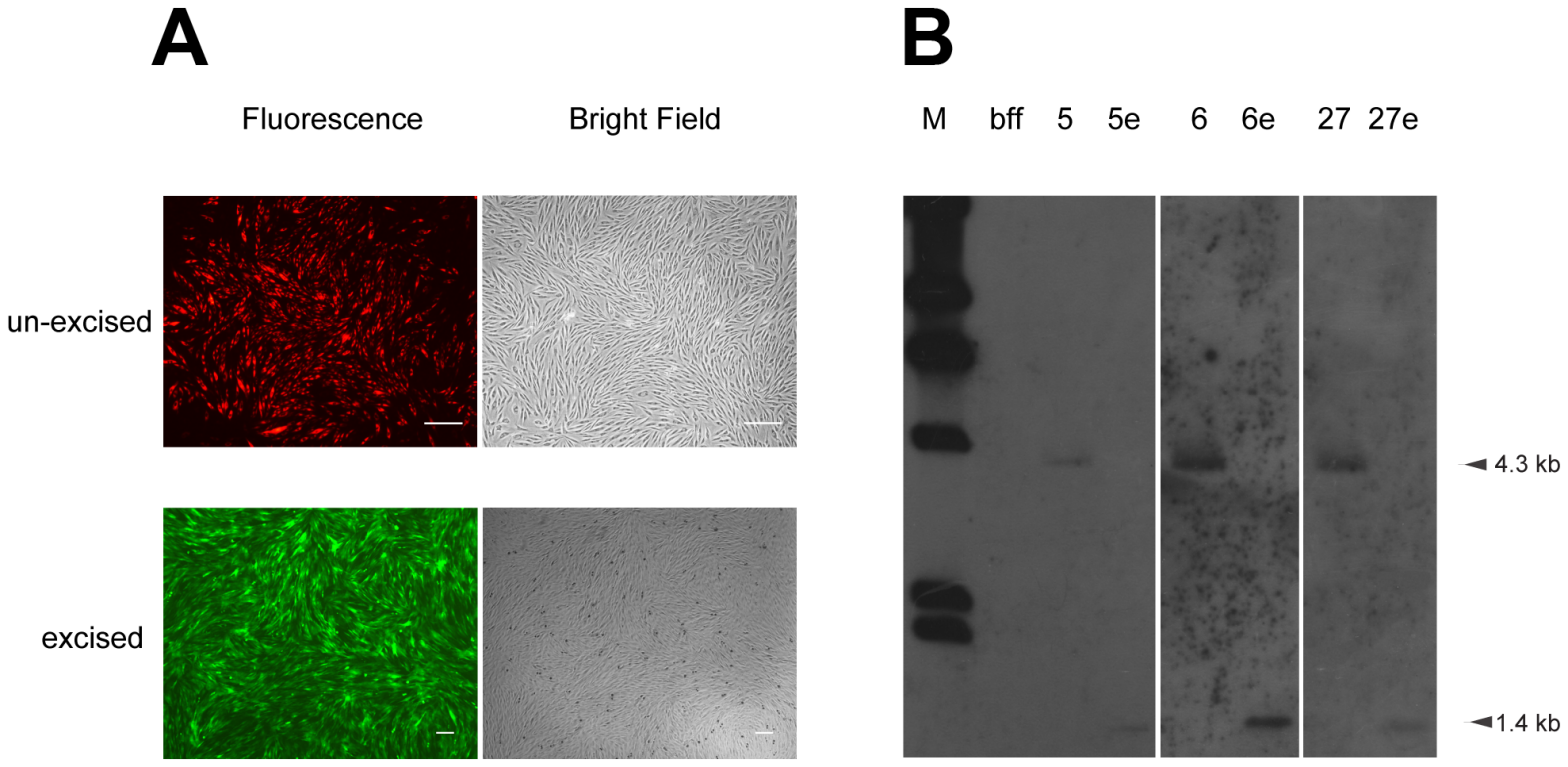
Identification of excision of the antibiotic selectable neoR marker in the transgenic cells. (a) Fluorescence phenotype of transgenic cells before and after Cre-mediated excision. The scale bar indicates 20 µm. (b) Southern blot analysis of three representative transgenic cell linesbefore and after Cre-mediated excision of the selectable marker gene using an *EGFP* probe. The un-excised clones (SC5, SC6 and SC27) carried a single integration of the transgene cassette (4.3 kb), whereas the excised clones (SC5E, SC6E and SC27E) also presented a single lower band (1.4 kb).

For simplicity and as a proof of concept, we focused on three clones, SC5, SC6 and SC27, each with a single integration site in the safe harbor. At day 5 after His-NLS-TAT-Cre protein transduction, these colony cells were prepared for flow cytometry by trypsinization and resuspension in PBS containing 10% fetal bovine serum. By visually tracking the phenotypic switch from red to green fluorescence, more than 70% GFP+ cells were easily detected (Fig. S2a) and isolated for clonal expansion by fluorescence-activated cell sorting (FACS) using a BD FACSAria. Excision of the *neoR* gene was verified by fluorescence visual detection (Fig. 3a) and Southern blotting (Fig. 3b). Moreover, the absence of either phiC31 integrase-encoding DNA or Cre recombinase-encoding DNA was demonstrated by PCR (Fig. S3a).

The integration sites of these excised transgenic cells were accurately confirmed by junction PCR (Fig. S3b). As expected, the integration sites of these excised cells remained the same as those of the un-excised ones. Thus, we tested the effect of the insertion and excision on the expression of two neighboring genes, *KIAA1486* and *IRS-1* genes, using qRT-PCR and compared the expression of these two genes with that in untransfected bovine fetal fibroblasts as the control (n = 3). We found no significant difference in the gene expression levels of *KIAA1486* and *IRS-1* in un-excised and excised transgenic cells, and untransfected bovine fetal fibroblasts (Fig. S3c), indicating that the insertion and excision did not alter the expression of flanking genes. In addition, chromosome spreads of metaphase cells of the selected clones were analyzed and revealed the correct chromosome number (2n = 58+ XX) and no major differences among un-excised and excised transgenic cells, and untransfected bovine fetal fibroblasts (Fig. S4). However, more refined cytogenetic techniques would be required to reveal more subtle chromosomal rearrangements that may occur in transgenic cells derived by this protocol. Such analyses will be carried out in the next study.

### Production of Antibiotic Selectable Marker Free Transgenic Embryos by SCNT

Antibiotic selectable marker free cells from different clones with different integration sites were used as nuclei donors for production of transgenic SCNT embryos. In addition, normal bovine fetal fibroblasts derived from the same fetus and at a similar passage number (passage 5–10) were used as a control to investigate the effect of transgenic procedures on the developmental potential of SCNT embryos. As shown in Table 2, no significant differences were observed in the cleavage and blastocyst formation rates among these antibiotic marker free clones and control groups.

**Table 2:** Summary of *in vitro* and *in vivo* development following SCNT with antibiotic selectable marker-free cells.

Donor cells*	Insertionsite	No.cultured	No.cleaved (%)‡	No. blastocysts (%)‡	No.recipients§	No. pregnancies (%)¶	No. calve born (%)||	No. calve survived
SC5E	BFF2†	475	372 (78.3±1.4)	129 (27.2±0.8)	41	7 (17.1)	3 (7.3)	1
SC6E	BFF2	781	615 (78.7±2.0)	210 (26.9±0.5)	67	12 (17.9)	5 (7.5)	4
SC27E	BFF2	602	473 (78.6±2.7)	163 (27.1±1.4)	52	9 (17.3)	4 (7.7)	3
SC10E	BFF4a	705	545 (77.3±2.4)	182 (26.4±0.4)	72	4 (5.6)	1 (1.4)	1
SC11E	BFF4b	603	472 (78.3±2.1)	160 (26.5±0.9)	51	5 (9.8)	2 (3.9)	1
SC2E	BFF13	457	353 (77.3±2.3)	117 (26.4±1.1)	46	2 (4.3)	–	–
SC4E	BFF19	812	627 (77.2±1.3)	215 (26.5±2.0)	68	4 (5.9)	2 (2.9)	1
SC1E	BFF22	786	615 (78.4±5.6)	209 (26.6±1.6)	67	5 (7.5)	1 (1.5)	1
SC16E	BFF25	411	324 (78.6±3.2)	104 (26.9±1.2)	42	2 (4.8)	–	–
Control	–	1699	1304 (76.9±5.9)	481 (28.3±2.1)	150	28 (18.7)	13 (8.7)	9

* Donor nuclei from antibiotic selectable marker-excised cells with single-copy integration of transgene into different locus, or from untransfected cells (control), were used for nuclear transfer experiments.

† BFF2 was evaluated as a genomic safe harbor according to the criteria articulated in a recently published study by Papapetrou et al.

‡ Values within one column are not significantly different (P>0.05).

§ Day-7 blastocysts were nonsurgically transferred to synchronized recipient cows with two embryos per recipient.

¶ No. pregnancies: number of pregnant recipients on day 90 d after embryo transfer/number of recipient cows.

|| Percentage of transferred recipients.

**Figure 4:**
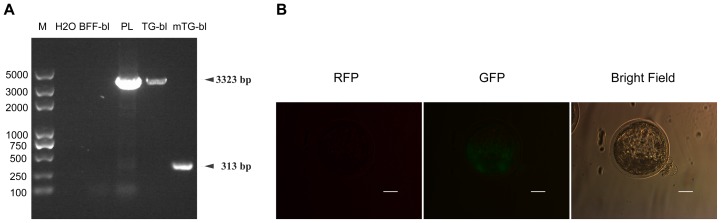
Identification of excision of the antibiotic selectable neoR marker in the transgenic embryo. (a) PCR analysis of single blastocyst to verify excision of the selectable marker gene in the excised transgenic cell derived blastocyst. BFF-bl represented untransfected bovine fetal fibroblast-derived blastocyst, was used as a negative control; PL (pARNG-HBD3 plasmid) was used as a positive control. TG-bl and mTG-bl were un-excised and excised colony derived blastocysts, respectively. (b) Fluorescence phenotype of the excised transgenic cell derived blastocyst. The scale bar indicates 200 µm. Only green fluorescence was observed.

PCR analysis of single blastocyst showed that a 3323 bp fragment could be amplified from the un-excised transgenic cell derived blastocyst but only a 313 bp fragment could be amplified from the excised transgenic cell derived blastocyst (Fig. 4a). The 3323 and 313 bp PCR fragments were sequenced. In addition, the fluorescence phenotype of excised transgenic cell derived blastocyst (Fig. 4b) confirmed that the selectable *neoR* marker gene had been successfully removed prior to SCNT.

### Generation of Cloned Transgenic Cattle that Express Human β-defensin-3

After SCNT, 1012 antibiotic selectable marker free transgenic blastocysts were transferred into 506 recipient cattle. The BFF2 site integrated clones showed similar pregnancy rate (17.1%–17.9%) and calf birth rate (7.3%–7.7%) with nontransgenic control (18.7% and 8.7%, respectively), while the pregnancy rate and birth rate of the other site integrated clones were lower than those of nontransgenic control (Table 2). A total of 18 calves were born, two calves died within a few hours after birth, and four calves died within 6 months after birth. Twelve calves survived and were healthy after weaning. As shown by PCR analysis, *human β-defensin-3* transgene was integrated into the genome of the 12 transgenic cattle (Fig. S5), but only five transgenic cattle, mTG1, mTG2, mTG3, mTG4 and mTG5, were lactating naturally when the observation started. Thus the data from subsequent experiments to be report just focus on these five transgenic cattle. Of the five cattle, mTG1 and mTG4 were generated from different clones with the same safe harbor integration, mTG2 was generated from BFF19 site integrated colony, mTG3 was derived from BFF22 site integrant, and mTG5 was from BFF4b site integrant. In addition, five unrelated non-transgenic, nonclone Holstein cow, matched by age and lactation, were chosen as control group. The breeding conditions were identical for the two groups.

**Figure 5:**
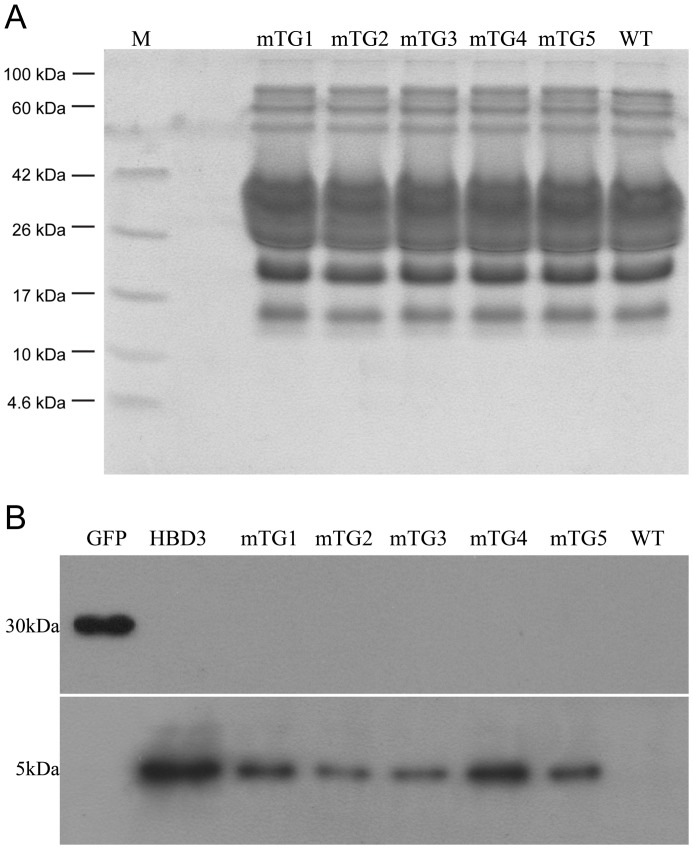
Protein analysis of milk from antibiotic selectable marker free transgenic cattle and non-transgenic cow. (a) Total proteins from transgenic cows mTG1–5 and non-transgenic animals were separated on a 10% Tricine-SDS-PAGE gel and visualized by Coomassie blue staining. Equal amounts of total milk protein were loaded onto each lane of the gel. (b) Western blot analysis of milk probed with an anti-human β-defensin-3 antibody or an anti-EGFP antibody. HBD3, commercial human β-defensin-3 (positive control); GFP, cell lysate of excised transgenic colony (positive control); WT, non-transgenic milk; and the other lanes, milk from transgenic cattle mTG1-mTG5.

Milk samples from transgenic and non-transgenic cattle were collected each month for 6 months during their natural lactation period. There were no significant differences in milk yield (P = 0.239) and percentage of fat, protein, lactose, and milk solids in the milk of transgenic and non-transgenic cattle (P = 0.793, 0.569, 0.696, and 0.976, respectively), as shown in Table 3. Milk proteins of the transgenic animals, as visualized on a polyacrylamide gel, appeared essentially identical to those from a non-transgenic Holstein cow (Fig. 5a). A single protein of the predicted size was immunologically reactive to antibodies against human β-defensin-3. The protein was observed in the milk of five transgenic cows, but not in that of non-transgenic cows (Fig. 5b). GFP was not detected in the milk of transgenic or non-transgenic cows (Fig. 5b). Human β-defensin-3 concentrations, as measured by ELISA, ranged from 3.9 to 10.4 µg/mL in milk of the five transgenic cows and no significant decline of human β-defensin-3 expression was observed during the natural lactation period of 6 months (Fig. S6). The lytic activity of milk samples from various transgenic cows was pre-estimated by an agar diffusion test (Fig. S7). The transgenic cows’ ability to resist infection by *S. aureus* and *E. coli* was tested by intramammary infusion of viable bacterial cultures. Of the mammary glands infused with *S. aureus*, 14 of 15 glands became infected in nontransgenic animals compared to 5 of 15 glands in transgenic animals (P = 0.001, Table 4); While of the mammary glands infused with *E. coli,* 13 of 15 glands became infected in nontransgenic animals compared to only 1 of 15 glands in transgenic animals (P = 0.001, Table 4). With the higher human β-defensin-3 expression level, transgenic cow mTG1 and mTG4, which derived from the evaluated safe harbor integrated transgenic cells, never became infected after *S. aureus* or *E. coli* infusion. Transgenic cow mTG2, mTG3 and mTG5, expressing lower level of human β-defensin-3, were infected twice, twice and once out of three infusions of *S. aureus*, respectively. Transgenic cow mTG2 was infected once out of three infusions of *E. coli.*

**Table 3:** Raw components of transgenic milk compared with conventional milk.

	Transgenic (n = 5)	Non-transgenic (n = 5)
Fat (g/100 mL)	4.07±0.32	4.01±0.35
Protein (g/100 mL)	3.45±0.24	3.60±0.52
Lactose (g/100 mL)	4.65±0.25	4.57±0.36
Solids (g/100 mL)	13.66±0.49	13.64±0.70

No significant differences were detected between transgenic group and non-transgenic group (P>0.05).

**Table 4:** Infection rate of *S. aureus* and *E. coli* infused into mammary glands of five transgenic and five non-transgenic lactating cows.

Cow*	*S. aureus*	*E. coli*	Overall
mTG1	0/3†	0/3	0/6
mTG2	2/3	1/3	2/6
mTG3	2/3	0/3	1/6
mTG4	0/3	0/3	0/6
mTG5	1/3	0/3	0/6
Non-tg (n = 5)	14/15	13/15	27/30

During each challenge experiment each gland was infused with *S. aureus*, *E. coli* or PBS, the fourth gland remained untreated. Infection was defined by the presence of viable *S. aureus* or *E. coli* in two consecutive milk samples. None of transgenic or nontransgenic glands infused with PBS became infected, so were the untreated transgenic and nontransgenic gland.

* Transgenic cows or non-transgenic cows were challenged three times at interval of two months during their natural lactation period.

† (Number of glands infected)/(number of glands infused).

## Discussion

In this study, we attempted to establish a rapid and safe procedure to generate antibiotic-resistant marker-free transgenic cattle. We generated safe harbor integrated bovine fetal fibroblast colonies in the presence of phiC31-NLS mRNA and employed a fluorescence double reporter system to sort antibiotic-resistant marker-free transgenic cells after cell-permeant Cre protein treatment. Our success of generating live cloned cattle using the antibiotic selectable marker free fibroblasts as nuclear donors in SCNT provided strong evidence that no severe damage was caused to the host cells using this method.

Because selectable marker elimination in plants was first reported in 1991 [27], marker-free technology has been already applied to many plant species such as rice [28], maize [29], [30], and tomato [31], [32]. With the development of transgenic animals, especially farm animals, removal of antibiotic selectable markers in transgenic animals has been recently brought to the forefront of research. Several antibiotic selectable marker free transgenic animals or donor cells prepared for SCNT has been established in various studies. Xu et al. [25] obtained two live cloned goats with removal of the selectable gene cassette. Wang et al. [26] successfully excised the *neoR* gene in transgenic cloned cattle. Generally, two steps are required to prepare antibiotic selectable marker free transgenic donor cells for SCNT: generation of transgenic animals with a *loxP*-flanked selectable marker and removal of the selectable marker gene from fibroblasts isolated from the generated transgenic animals by Cre recombinase. The two-step manipulation does not affect the developmental competence of recloned preimplantation embryos [25], [26], however, it is costly and time consuming to prepare *loxP*-flanked transgenic animals (almost a year, take cattle for example) and requires labor-intensive work to identify complete excision events among randomly picked colonies.

In our study, the fluorescence double reporter was used to monitor Cre-mediated recombination in living cells before and after Cre recombination by expression of two different fluorescent proteins. Before Cre-mediated excision, transgenic cell colonies were easily generated by the design of both a selectable marker and fluorescent reporter. The *DsRed* reporter was employed to exclude either few false positive cells that were insensitive to G418 or stably-transfected colonies that integrated in a transcriptionally silent region on chromosomes. Subsequently, Cre recombination resulted in replacement of *DsRed* expression with *EGFP* expression. Cells showing both GFP+ and RFP− were considered as cells with the selectable marker completely removed and were sorted by FACS for SCNT. Finally, the absence of the selectable marker gene in both sorted GFP+ cells and blastocysts derived from such cells was verified by Southern blot and PCR, thereby providing strong evidence that the Cre/loxP system combined with fluorescence double reporter offers a highly efficient and reliable tool to remove selectable maker genes completely and visually.

To avoid unwanted translocation events caused by multiple *loxP* sites on different chromosomes [33], the phiC31 integrase system was employed to generate a single copy of *loxP*-flanked *neoR*. PhiC31-mediated integration events were analyzed by absolute quantity PCR to identify single integrants that were used for subsequent steps. In this study, at least 24% (11/46) of all G418-resistant colonies showed a single integration of the pARNG-HBD3 plasmid. This fraction is superior to that of single-copy integrants obtained with other non-viral methods such as PB transposon-based approaches (15%) [34], [35]. Very recently, a profile of bovine native integration sites for phiC31 integrase has been well described [36]. To evaluate the feasibility of our approach for the bovine genome, we re-analyzed previous DNA sequence data of phiC31 integration sites in bovine cells [36]–[38], according to all of the safe harbor criteria proposed by Papapetrou et al. [21]. This analysis revealed that of the 33 sequenced native pseudo *attP* sites, 17 were intergenic, representing 52%, and 5 of the intergenic sites met the criteria of genomic safe sites, representing 15% of the total integration sites (Table S1). Notably, in our study, 13 out of 46 colonies of the integrase group showed integration at BFF2 site in intergenic region between *KIAA1486* and *IRS-1*, which met all of the criteria of a genomic safe harbor [21]. The BFF2 site has 33% sequence identity with wild-type *attP*, while other integration sites that have the similar or even better homology did not show such integration frequency. It is possible that some integration sites that have a lower level of sequence identity have a more highly preferred genomic context. Thus, contextual factors, such as repetitive sequences surrounding the integration sites, may play a strong role in site selection as well as primary sequence.

Another advantage of the integrase system is that the distance of the single insertion from genes of known and unknown function can be determined precisely [39]. Hence, it is possible to determine whether the intergenic insertion affects the expression of flanking genes. We tested three colonies in which the integration occurred in the intergenic region on chromosome 2, both before and after Cre-mediated excision. qRT-PCR showed that the expression of neighboring genes was unaffected even after removal of the selectable marker by Cre treatment. At the very least, using the integrase system, transgenic donor cells can be prepared in a rather safe manner.Recently, a similar site-specific recombinase strategy was reported to create reprogramming factor-free induced pluripotent stem (iPS) cells [20]. However, delivery of phiC31 and Cre expression plasmids may result in unwanted insertion, and constitutive expression of phiC31 integrase [40], [41] or Cre recombinase [42]–[44] can induce cytotoxicity and genotoxicity. In our study, we used phiC31 mRNA for site-specific integration and cell-permeant Cre protein for removal of the selectable marker in a highly efficient and safe manner. Furthermore, no abnormal chromosome numbers were found in transgenic cells before or after cell-permeant Cre treatment. There is evidence indicating that the extracellular concentration of cell-permeable Cre protein can be precisely optimized to induce recombination, while causing no apparent toxicity [45], [46]. Therefore, this controllable and mild method provides the possibility for a second genomic manipulation immediately after the stably transfected cells are generated, which saves time and minimizes expenses compared with that using the traditional two-step method as discussed above.

The development ability of the transgenic embryos or non-transgenic controls in this study was somewhat lower than that in others’ work [47]–[49]. Numerous factors, such as the state and source of donor cells [49]–[51], cytoplast source and quality [52], methods of manipulation and activation [53], [54], and embryo culture conditions [55], could have an impact on the efficiency of SCNT embryo development and caused the difference. In this study, no significant difference was found in cleavage rate, blastocyst rate, pregnancy rate and birth rate between the experimental (BFF2) and non-transgenic group, which indicated that the developmental ability of the antibiotic selectable maker free transgenic embryos we produced was not compromised by using this procedure. However, other transgenic embryos derived from the non-safe harbor integrated transgenic clones we produced showed relatively lower developmental competence compared with the control group, which might suggest that integration site of transgene is crucial in this procedure, some undetermined integration sites might be detrimental to the nuclear donor cell.

Human β-defensin-3, a kind of antimicrobial peptide, is widely expressed in many tissues [56], [57]. Human β-defensin-3 has broad-spectrum antimicrobial activity against bacteria, fungi, and enveloped viruses, and has an important role in immunity [58]. In previous studies, transgenic livestock expressing some kinds of antimicrobial peptides in milk inhibited bacterial pathogens causing mastitis [59]–[61]. However, both lysostaphin [61] and lysozyme [62] were effective against *S. aureus* but not *E. coli*, and other human β-defensins exhibited less activity against gram-positive bacteria, including *S. aureus*, than against gram-negative bacteria [63]. While human β-defensin-3 showed antibacterial activity against both *S. aureus* and *E. coli* at very low concentrations [56],[62] and exhibited less salt sensitive than other human β-defensins [64]. Therefore, *human β-defensin-3* might be a candidate gene for enhancing mastitis resistance. By simple evaluation of transgenic milk samples from five transgenic cows, which were lactating normally in the observation period, the bacteriolytic activity of the milk was confirmed. Transgene expression levels in the animals reported here were similar to those found in some livestock bioreactors [61], but less than those found in other livestock bioreactors [47], [60], [65]. Some reasons might cause this difference. Firstly, more regulatory elements in transgenic vector may lead to high expression level, for example using bacterial artificial chromosome (BAC) [65] or insulators [60]. Secondly, the different levels of expression among transgenic cattle may relate to the copy numbers of the transgene [66]. Thirdly, the signal peptide of human β-defensin-3 we used in this study may have lower secretory capacity than that of others, such as human growth hormone [67]. In addition, many factors of the SCNT procedure, such as the quality of the donor cells, the nuclear reprogramming, could also lead to different levels of expression [68]–[70]. The exact mechanisms remain to be determined, however.

However, the human β-defensin-3 expression levels remained stable during the natural lactation period of 6 months, and milk from these transgenic cows repressed the growth of both *S. aureus* and *E. coli*, the most prevalent species of gram-positive and gram-negative bacteria that induce clinical mastitis [71]. The transgenic cows’ ability to resist infection by *S. aureus* and *E. coli* was confirmed by intramammary infusion of viable bacterial cultures. The infection rate between nontransgenic animals and those expressing human β-defensin-3 provided strong evidence that the transgene product conferred a protective effect. Because of the small transgenic sample size it is not possible to make any statistical inferences regarding to the infection frequency and human β-defensin-3 milk concentration. However, the data are suggestive. The three cows with the lower human β-defensin-3 expression level had the higher *S. aureus* infection rate (2/3, 2/3 and 1/3, respectively), and impressively, only one out of all fifteen *E. coli* infused transgenic glands became infected. Notably, the higher expressing transgenic cows, which derived from the evaluated safe harbor integrated transgenic cells, never became infected after *S. aureus* or *E. coli* infusion.

Interestingly, only human β-defensin-3, but not GFP, was detected in the transgenic milk, which might be caused by the fluorescent protein lacking the signal peptide. GFP, as a fluorescent reporter to monitor the excision of the selectable marker, remained constitutively expressed in transgenic donor cells generated by this protocol. Multiple studies have used the green fluorescent protein gene transfected donor cells and subsequently used for NT to create bovine embryos and calves [72]–[75]. The positive GFP embryos demonstrate easily detectable bright fluorescence and no genetic screening (PCR) is required prior to transfer. Recently, it has been reported that GFP expression does not appear to have unexpected or undesirable effects on transgenic animals, and it is unlikely that the welfare of transgenic animals is compromised [76]. Thus far, in our study, it has not been found that the expression of the transgene influences the welfare and behavioral phenotype of the generated antibiotic selectable marker free transgenic cattle. Thus, we considered that *EGFP* is a useful marker for screening preimplantation embryos to increase the overall efficiency of transgenic animal production, and its non-secreting character makes it an acceptable tool for generation of an antibiotic selectable marker free transgenic mammary bioreactor.

In conclusion, we performed safe harbor integration in the bovine genome using phiC31 integrase and the combination of the Cre/loxP system with a fluorescent double reporter to remove the selectable marker efficiently. The non-viral protocol (Fig. S8) offers a rapid and safe alternative to produce antibiotic selectable marker free transgenic large animals, thereby making it a valuable tool for promoting the healthy development of transgenic large animals, and their recombinant products may be more easily accepted by the public.

## Materials and Methods

### Ethics Statement

The experimental procedure was approved by the Animal Care Commission of the College of Veterinary Medicine, Northwest A&F University. Bovine ovaries from slaughtered mature cows were collected from Tumen abattoir, a local abattoir in Xi’An, China. A newborn female Holstein calf was obtained for nuclear donor cell cultures and Beef-breed Angus cows were obtained for recipient animals from Yangling Keyuan Cloning Co., Ltd., PR China.

### Chemicals

All chemicals and reagents were purchased from Sigma-Aldrich (St. Louis, USA) unless specifically stated otherwise. Disposable, sterile plasticware was purchased from Nunclon (Roskilde, Denmark).

### Plasmid Construction

Primer sequences, restriction sites and templates are depicted in Table S2.

For construction of the universal fluorescent double reporter plasmid, pARNG, the pEGFP-C1 plasmid (Clontech, Mountain View, CA) was digested with BamHI/BglII and self-ligated to remove the original multiple cloning sites. To construct pAttB-eGFP, this vector (pEGFP-B2) was digested with AseI and ligated to a 287 bp *attB* fragment generated using splicing by overlap extension (SOE) PCR with attB1_F∼attB4_R primers listed in Table S2. A *loxP*-flanked MCS was obtained by SOE-PCR with LoxPMCS F1∼LoxPMCS R2 primers and subsequently cloned into pAttB-eGFP. The pAttB-LoxP2-eGFP plasmid was digested with SpeI/AflII and ligated to a new KanR/neoR fragment generated with Kneo_F and Kneo_R primers. Also, an additional SV40 poly(A) was obtained and cloned downstream of the KanR/neoR expression cassette. This vector (pAL2G-neopA) was digested with MluI/EcoO109I to replace the original KanR/neoR expression cassette with another synthetic MCS for subcloning. To construct pDsRed1-B2, the pDsRed1-C1 plasmid (Clontech) was digested with BamHI/BglII and self-ligated to remove the multiple cloning sites. Then the final pARNG plasmid was generated by cloning a 900 bp *DsRed* ORF and an SV40 poly(A) sequence derived from pDsRed1-B2 into the pANG-MCS plasmid. For subcloning of the *human β-defensin-3* expression cassette into pARNG, the *human β-defensin-3* genomic gene (1 kb) cloned from human placenta, as well as the CSN2 promoter (3.7 kb) and 3′ flanking region (0.6 kb) cloned from the Holstein female bovine genome were ligated to pARNG in correct order as shown in Figure 1.

To transcribe phiC31 mRNA *in vitro*, pcDNA3.1-Int plasmid was constructed by cloning the phiC31 integrase ORF into pcDNA3.1(+) (Invitrogen, Carlsbad, California) with an addition of a Kozak site and an NLS sequence using IVTInt_F and IVTInt_R primers. The pcDNA3.1-mtInt plasmid was constructed by deleting a 537 bp in-frame fragment in the integrase gene using PmlI (New England Biolabs, Beijing, China). The deletion reduced the polypeptide length from 621 amino acids (68 kDa) to 442 amino acids (48 kDa) and encompassed the entire DNA binding region of the protein to render it inactive [41].

The pET-28a(+)-His-NLS-TAT-Cre plasmid was constructed for prokaryotic expression of recombinant Cre protein. A NLS-TAT-encoding fragment was generated by annealing the NT_F and NT_R primers. This fragment was cloned into pET-28a(+) (Novagen, Madison, WI) via the NdeI and SacI restriction sites, resulting in pET-28a(+)-His-NLS-TAT. To obtain pET-28a(+)-His-NLS-TAT-Cre, a Cre-encoding PCR product was generated from the pCAG-Cre-IP template [77] using CreTAA_F and CreTAA_R primers. This fragment was subsequently cloned into pET-28a(+)-His-NLS-TAT via SacI and XhoI sites.

### Preparation of phiC31 mRNA

pcDNA3.1-Int or pcDNA3.1-mtInt plasmid DNA was linearized by PmeI (New England Biolabs) downstream of the coding region as the template (1 µg). PhiC31 integrase or mutant integrase mRNA was transcribed *in vitro* using a mMESSAGE mMACHINE® T7 mRNA transcription kit (Ambion, Austin, TX), and the polyadenylation reaction was performed using a Poly(A) Tailing Kit (Ambion). Purification of the tailed mRNA was performed using a MEGAClear™ kit (Ambion). All procedures followed the kit protocols. mRNA concentrations were determined and diluted to about 500 ng/µL for electroporation, and stored at −80°C.

### Cell Culture and DNA/RNA Co-transfection

Bovine fetal fibroblasts were isolated from a 50–60-day-old Holstein female fetus (Yangling Keyuan Biotechnology Inc) by disaggregation of the body without the head and viscera, followed by culturing in Dulbecco’s modified Eagle’s medium (DMEM; GIBCO, Carlsbad, CA) supplemented with 10% fetal bovine serum (FBS; GIBCO) at 38.5°C in a humidified atmosphere with 5% CO_2_. At confluency, bovine fetal fibroblasts were collected by trypsinization for passaging or cryopreservation.

To co-electroporate the *human β-defensin-3* donor plasmid and phiC31 mRNA, 1×10^6^ passage 3 cells were re-suspended in 0.2 mL diluted electroporation working buffer [electroporation buffer (120 mM KCl, 0.15 mM CaCl_2_, 10 mM K_2_HPO_4_, and 5 mM MgCl_2_) : opti-MEM (GIBCO) = 3∶1 (v/v) ] after being washed twice in opti-MEM and once in ice-cold PBS. Then, 5 µg pARNG-HBD3 plasmid DNA and 1 µg phiC31 integrase or mutant integrase mRNA were added to the cells. Electroporation was performed with a BTX ECM2001 set to a single pulse of 2 ms at 510 V. At 24 h after electroporation, ∼30,000 cells were diluted in a 10-cm dish containing fresh medium supplemented with G418 (600 mg/mL) for selection of stably transfected cells. Individual cell colonies were isolated at 8–12 days after dilution culture.

### Quantitative RT-PCR Analyses

Real-time PCR was performed using SYBR Premix Ex TaqTM (TaKaRa, Dalian, China) and a StepOne Plus thermocycler (Applied Biosystems) with the following parameters: 95°C for 10 sec, followed by 40 cycles at 95°C for 5 sec and 60°C for 30 sec. For RT-PCR, total RNA was extracted from each sample using TRIZOL reagent (Invitrogen), and reverse transcription was performed to generate cDNA using a PrimeScript™ RT Reagent Kit (TaKaRa). The RFP gene primers used in absolute quantitative PCR were 5′-GCCACAACACCGTGAAGCTGAA-3′ (forward) and 5′-ACACCTTGGAGCCGTACTGGAA-3′ (reverse). The GAPDH gene, as a reference gene in relative quantitative RT-PCR, was amplified using the following primers: 5′-TCAACGGGAAGCTCACTGG-3′ (forward) and 5′-CCCCAGCATCGAAGGTAGA-3′ (reverse). For each DNA and cDNA sample, target and reference genes were amplified independently on the same plate and in the same experimental run in triplicate. PCR specificity was confirmed by gel electrophoresis on a 2.5% agarose gel and by a single peak in the melting curve. For relative quantitative RT-PCR, the amount of target normalized to the reference was calculated by the 2^−ΔΔCt^ method.

### Establishment of the Absolute Quantitative Standard Curve

To examine the copy number, generation of an absolute quantitative standard curve was necessary. A series of standard samples containing 0.5, 1, 2, 4, 8, and 16 copies of the RFP gene were prepared as described previously [66] by mixing the wild-type genome of an Holstein cow with the pARNG-HBD3 plasmid. To prepare a standard sample containing one copy of the RFP gene, the quantity of the plasmid mixed with genomic DNA was calculated by the following formula: 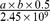 (“a” represents the size of plasmid). The absolute quantitative standard curve was drawn by plotting Ct values against the log of RFP gene copies of the corresponding standard samples. The parameters of the standard curve were: log2N = –0.9568 Ct+25.335 (R^2^ = 0.9887, P<0.001).

### Half-nested Inverse PCR and Integration Site Analysis

Genomic DNA was isolated from stably transfected cell colonies by phenol/chloroform extraction and ethanol precipitation. Half-nested inverse PCR was performed to identify the pseudo *attP* sites as described previously [38]. Briefly, 5 µg genomic DNA was digested with the isocaudamer enzymes NheI and SpeI (New England Biolabs). After extraction with phenol/chloroform and ethanol precipitation, digests were ligated with a DNA Ligation Kit (TaKaRa) at 4°C overnight in a total reaction volume of 50 µL. Ligated DNA was extracted with phenol/chloroform and ethanol precipitation, and suspended in 25 µL nuclease-free water. Primary PCR was performed with HNI_F1 and HNI_R primers that were designed against *attL* sites (Table S2) as follows: 94°C for 5 min, followed by 30 cycles of 94°C for 30 s, an annealing temperature gradient of 51–61°C for 45 s and 72°C for 5 min, and then 72°C for 10 min. One microliter of the PCR product was used as a template in the secondary reaction. The secondary PCR was performed with the HNI_F2 and HNI_R primers (Table S2). The PCR conditions were the same as those for the primary PCR except for an annealing temperature gradient of 50–60°C. PCR products were run on agarose gels and the resulting bands were excised. The purified product was cloned into a pMD19-T vector (TaKaRa) and sequenced with M13 and reverse M13 primers. Sequences were examined by BLAST searching of the bovine genome databases (http://www.ncbi.nlm.nih.gov/genome/seq/BlastGen/BlastGen.cgi?taxid=9913).

To analyze repetitive sequences surrounding pseudo *attP* sites, genomic sequences flanking phiC31 integration sites (540 bp) were aligned to bovine repeat sequences using the Repeat Masker Web Server (http://www.repeatmasker.org, Institute for Systems Biology), microRNA around the pseudo *attP* sites was searched using miRBase 18.0 (http://www.mirbase.org/), a cancer related gene and UCRs were obtained from statistics reported previously [21], and the data were downloaded from http://users.soe.ucsc.edu/~jill/ultra.html.

### Preparation of Cre Recombinant Protein and Cre Protein Transduction

pET-28a(+)-His-NLS-TAT-Cre plasmids were used to transform *E. coli* strain BL21 (DE3) for IPTG-inducible expression of His-tagged Cre protein. Bacteria were inoculated at 1∶50 into 500 mL Luria Bertani (LB) medium containing 50 µg/mL kanamycin and grown at 37°C until an OD_600_ of 0.7. Overexpression was induced by addition of IPTG at a final concentration of 0.7 mM, followed by incubation for 8 h at 28°C. Cells were harvested by centrifugation and stored at –20°C. Cell pellets were thawed by resuspension in 20 mL binding buffer (50 mM NaH_2_PO_4_, pH 8.0, 300 mM NaCl, and 10 mM imidazole). Cleared lysate was obtained after incubation with 1 mg/mL lysozyme and 5 U/mL benzonase (Novagen) and centrifugation for 20 min at 12,000×*g* and 4°C. The supernatant was filtered through a 0.45 mm filter (Millipore, Billerica, MA), subsequently added to binding buffer and passed through a His60 Ni Gravity Flow Column (Clontech) to bind the His-tagged Cre protein to the Nickel column. The column was washed with 10 column volumes of His Ni wash buffer (50 mM NaH_2_PO_4_, pH 8.0, 300 mM NaCl, and 20 mM imidazole). The His-tagged Cre protein was eluted with 10 column volumes of elution buffer (50 mM NaH_2_PO_4_, pH 8.0, 300 mM NaCl, and 250 mM imidazole) and then dialyzed against opti-MEM for immediate use or storage at –80°C. Protein concentrations were measured by a BCA Protein Assay Kit (Beyotime, Jiangsu, China).

For Cre transduction experiments, 2 × 10^6^ cells were seeded on a 24-well plate and grown for 24 h or until 90% confluency. Cre protein was dialyzed against opti-MEM and sterilized by filtration using a 0.22 µm filter (Millipore, Billerica, MA). Cells were incubated for 24 h in medium containing 100 µg/mL His-NLS-TAT-Cre protein. After transduction, cells were washed with PBS and cultured for an additional 72 h in normal medium before by flow cytometric analysis and FACS.

### Chromosome Analysis

The number of chromosomes in untransfected bovine fetal fibroblasts, and un-excised and excised transgenic cell colonies was determined by Giemsa staining. Slides were prepared by standard techniques. The appropriate spreads were photographed and used to count the number of chromosomes according to the size and position of the centromere.

### Southern Blot Analysis

Ten micrograms of genomic DNA from untransfected cells or stably transfected cell colonies was digested overnight with BsrGI and XhoI (New England Biolabs), and resolved by agarose gel electrophoresis. After transfer and UV crosslinking onto a Hybond N+ nylon membrane (Roche, South San Francisco, CA), the DNA was hybridized with an *EGFP* probe generated by a DIG High Prime Labeling and Detection Starter Kit II (Roche). The primers sequences were 5′-AAGTTCATCTGCACCACCG-3′ (forward) and 5′-TGCTCAGGTAGTGGTTGTCG-3′ (reverse).

### SCNT, Activation, and Culture of SCNT Embryos

SCNT, activation of reconstructed embryos, and culture of SCNT embryos were performed as described previously [78]. Briefly, matured oocytes were enucleated using a 20 mm inner diameter glass pipette to remove the first polar body and a small amount of surrounding cytoplasm. Successful enucleation was confirmed by Hoechst 33342 staining. A single disaggregated donor cell was injected into the pre-vitelline space of an enucleated oocyte. The oocyte-cell fusion was performed using a pair of platinum electrodes connected to a micromanipulator in microdrops of Zimmermann’s fusion medium, and a double electrical pulse of 35 V for 10 ms was used for fusion. Reconstructed SCNT embryos were kept in synthetic oviductal fluid (SOFaa) containing 5 mg/mL cytochalasin B for 2 h until activation. The mSOF medium was prepared according to a formula described previously [79] and supplemented with 8 mg/mL bovine serum albumin, 1% MEM non-essential amino acids and 2% BME essential amino acids. Activation of reconstructed embryos was performed in 5 mM ionomycin for 4 min followed by 4 h exposure to 1.9 mM dimethynopyridine in SOFaa. After activation, embryos were cultured in G1.3/G2.3 sequential media (Vitrolife AB, Gothenburg, Sweden). Droplets of 150 µL G1.3 were prepared in a 35-mm cell culture dish under mineral oil and equilibrated for 2 h before loading the embryos (20 embryos/microdrop). Embryos were transferred to G2.3 droplets on day 3 of culture (day 0 being the day of SCNT).

### Western Blot Analysis

Daily milk weights were recorded and milk sample was collected once a month for 6 months. The percentages (w/vol) of fat, protein, lactose, and solids were determined using a MilkoScan 6000 (Foss, Hillerod, Denmark). Milk samples and standards were separated by 10% Tricine-SDS-PAGE and electrotransferred onto polyvinylidene fluoride membranes (Millipore, Billerica, MA) for western blot analysis using a standard protocol. A rabbit polyclonal antibody against human β-defensin-3 (1∶1000, Sigma) and horseradish peroxidase-conjugated goat anti-rabbit IgG (1∶1000, Beyotime) were used to detect human β-defensin-3, and a mouse monoclonal antibody against EGFP (1∶5000, Clontech) and horseradish peroxidase-conjugated goat anti-mouse IgG (1∶1000, Beyotime) were used to detect GFP. Positive controls were commercial human β-defensin-3 (Sigma) for detection of human β-defensin-3, and a cell lysate of excised colonies for detection of GFP. The negative control was non-transgenic milk. Human β-defensin-3 milk concentration was determined by ELISA as per manufacturer’s directions (USCNK Life Sciences, Wuhan, China).

### Agar Diffusion Test

The disc diffusion method was performed with LB agar (Sigma). *S. aureus* (ATCC 25923) and *E. coli* (ATCC 25922) at mid-log phase (A_600_<0.6–0.7) was mixed with 20 mL solid culture medium containing 1.5% agar. Each sample was placed on a sterile, quantitative filter paper disc (6 mm in diameter) on a plate, and incubated for 24 h at 37°C. The results were assessed by inhibition zones around disc paper. Milk sample from a non-transgenic cow were placed on the filter paper disc as a negative control. The experiment was repeated three times.

### Challenge Study

Forty-eight hours before initiating the bacterial challenges, health of the animals was assessed by differential leukocyte and milk somatic cell counts to verify that they were within normal ranges. Milk from each quarter, cultured overnight also had to be free of bacterial growth for the animal to be included in the study. Since the udder contains four glands that function independently of one another, it is possible to expose a cow to multiple treatments, as was done in these experiments. After the morning milking, an aseptic milk sample was collected from each of the four glands before infusing 2 mL of different bacterial cultures of *S. aureus* (40±2 c.f.u/mL, diluted with PBS) or *E. coli* (40±3 c.f.u./mL, diluted with PBS) into one of two contralateral quarters via the streak canal. A third quarter received a similar infusion of sterile PBS only, and the fourth gland remained no infusion. Treatments were assigned such that no gland received the same strain in subsequent trials. The milk samples were taken at 12-h intervals or more frequently throughout the study. Milk samples (20 µL) were plated on blood agar and MacConkey agar incubated at 37°C for 18 to 24 h. An infection was confirmed by the presence of viable *S. aureus* growing on blood agar plates or *E. coli* growing on MacConkey agar in two consecutive milk samples.

### Statistical Analysis

Each experiment was performed at least three times. All data were analyzed using SPSS 20.0 statistical software (IBM Corporation, Somers, NY, USA). Data were tested by one-way ANOVA and least-significant difference tests, and reported as the mean ± SEM. For all analyses, P<0.05 was considered significant.

## Supporting Information

Figure S1 **Identification of transgenic colonies in the integrase group and the mutant integrase group.** *AttB* cleavage assay of the phiC31 integrase (a) and the mutant integrase (b) groups. All RFP+ colonies were subjected to the PCR test for the full-length *attB* site. A band of 426 bp for the un-cleaved *attB* site indicated non-site-specific integration. Genomic DNA from untransfected bovine fetal fibroblasts was used as a negative control, and PL (pARNG-HBD3) was used as a positive control. (c) Copy number assay of the 23 *attB*-cleaved colonies by absolute quantity PCR. Black columns represent a transgenic cell colony with a single copy integration, and gray columns represent double copy integration. Three or more copy integration events were not detected. Error bars denote SEM. (d) All 46 G418-resistant colonies were subjected to junction PCR with primers specific for the safe harbor and adjacent *attB* site. Genomic DNA from untransfected bovine fetal fibroblasts was used as a negative control.
(TIF)

Figure S2 **FACS analysis after His-NLS-TAT-Cre protein transduction into three RFP+ cells lines.** At Day 5 after protein transduction, cells were trypsinized and resuspended in PBS containing 10% FBS, and then analyzed for RFP and GFP expression by flow cytometry. More than 70% of the transduced cells were GFP+ as shown by flow cytometry. BFF cells were untransfected control.
(TIF)

Figure S3 **Further analysis of antibiotic selectable marker free transgenic cells.** (a) PCR to test for pCMVInt and pCAG-Cre-IP showing the absence of either phiC31 integrase or Cre recombinase encoding DNA in established excised transgenic colonies. Genomic DNA from untransfected bovine fetal fibroblasts was used as a negative control, and PL1 (pCMVInt plasmid) and PL2 (pCAG-Cre-IP plasmid) were used as positive controls. (b) Verification of the genomic integration sites of the established excised cells by junction PCR using pairs of the respective genomic and plasmid-binding primers. (c) Relative real-time RT-PCR analysis of *KIAA1486* and *IRS-1* gene expression in un-excised and excised transgenic cells compared with that in untransfected bovine fetal fibroblasts normalized to 1. Error bars denote SEM.
(TIF)

Figure S4 **Chromosome counts.** Metaphase spreads of un-excised and excised transgenic cells were counted and compared to untransfected bovine fetal fibroblasts.
(TIF)

Figure S5 **PCR analysis of transgenic cattle.** M, DNA ladder; WT, non-transgenic cattle; and PL, positive control from the constructed vector; line 1–12, marker-free transgenic cattle mTG1-12.
(TIF)

Figure S6 **Human β-defensin-3 concentration during the first lactation period of transgenic cows.** Milk was collected once a month for 6 months. Error bars denote SEM.
(TIF)

Figure S7 **Lytic activity of human β-defensin-3 and milk samples from transgenic cows against** ***S. aureus*** **and** ***E. coli*** **.** The small circles (6 mm in diameter) consist of quantitative filter paper with 10 µL of test sample or skim milk from non-transgenic cows (control). The larger circles are the inhibition zones. A, commercial human β-defensin-3; B–F, milk samples from transgenic cows mTG1–5; and G, milk samples from non-transgenic cows (control). The bar indicates 10 mm.
(TIF)

Figure S8 **Generation of antibiotic selectable marker free transgenic cells using site-specific recombinase.** To generate competent transgenic donor cells for SCNT efficiently, five main steps were used in our study as follows. Step 1: Co-electroporation with the transgene incoming plasmid and phiC31 integrase mRNA; Step 2: Generation of G418-resistant and RFP+ colonies; Step 3: Identification (*attB* cleavage assay → copy number analysis of colonies with cleaved *attB* site → integration site analysis of single-copy integrants) and proliferation; Step 4: Cre protein transduction into “safe harbor” integrated colonies; Step 5: Sorting cells showing both GFP+ and RFP− by FACS for SCNT.
(TIF)

Table S1 **PhiC31-mediated integration into the bovine genome.** Pseudo *attP* sites in Italian were from statistics reported in several other papers, the rest ones were found in our study. The pseudo sites presented in bold met all the safe harbor criteria in Papapetrou et al.
(XLSX)

Table S2 **Primers used for SOE-PCR, gene amplification, qRT-PCR and the detection of pCMVInt and pCAG-Cre-IP.**
(DOCX)
