# Supplementary figures and images for: A Site-Specific Recombinase-Based Method to Produce Antibiotic Selectable Marker Free Transgenic Cattle
Source: PLoS One. 2013 May 1;8(5):e62457. doi: 10.1371/journal.pone.0062457 (PMC3641042; doi:10.1371/journal.pone.0062457)

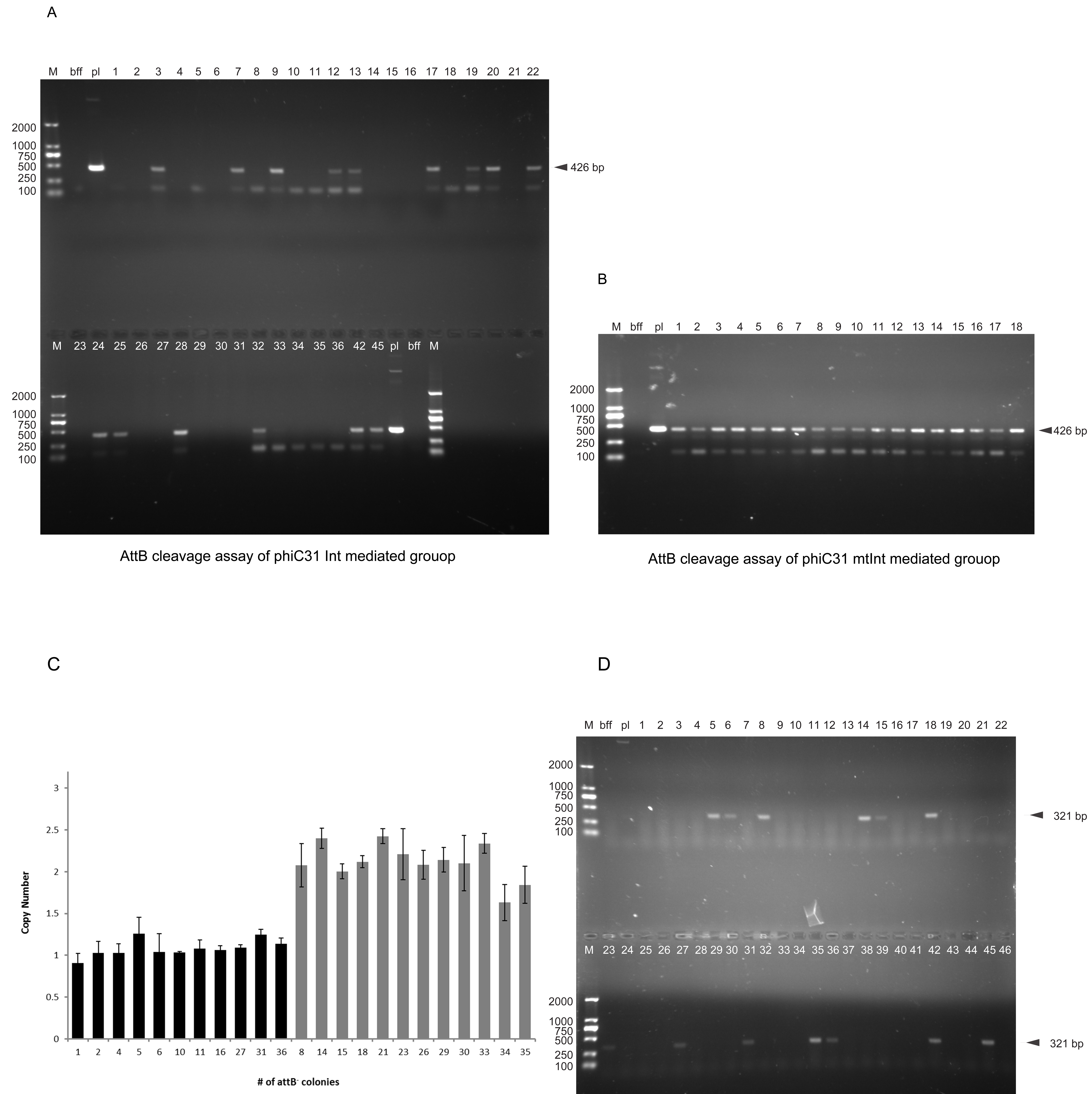

Supplement: Figure S1 — Identification of transgenic colonies in the integrase group and the mutant integrase group. AttB cleavage assay of the phiC31 integrase (a) and the mutant integrase (b) groups. All RFP+ colonies were subjected to the PCR test for the full-length attB site. A band of 426 bp for the un-cleaved attB site indicated non-site-specific integration. Genomic DNA from untransfected bovine fetal fibroblasts was used as a negative control, and PL (pARNG-HBD3) was used as a positive control. (c) Copy number assay of the 23 attB-cleaved colonies by absolute quantity PCR. Black columns represent a transgenic cell colony with a single copy integration, and gray columns represent double copy integration. Three or more copy integration events were not detected. Error bars denote SEM. (d) All 46 G418-resistant colonies were subjected to junction PCR with primers specific for the safe harbor and adjacent attB site. Genomic DNA from untransfected bovine fetal fibroblasts was used as a negative control. (TIF) [file pone.0062457.s001.tif]

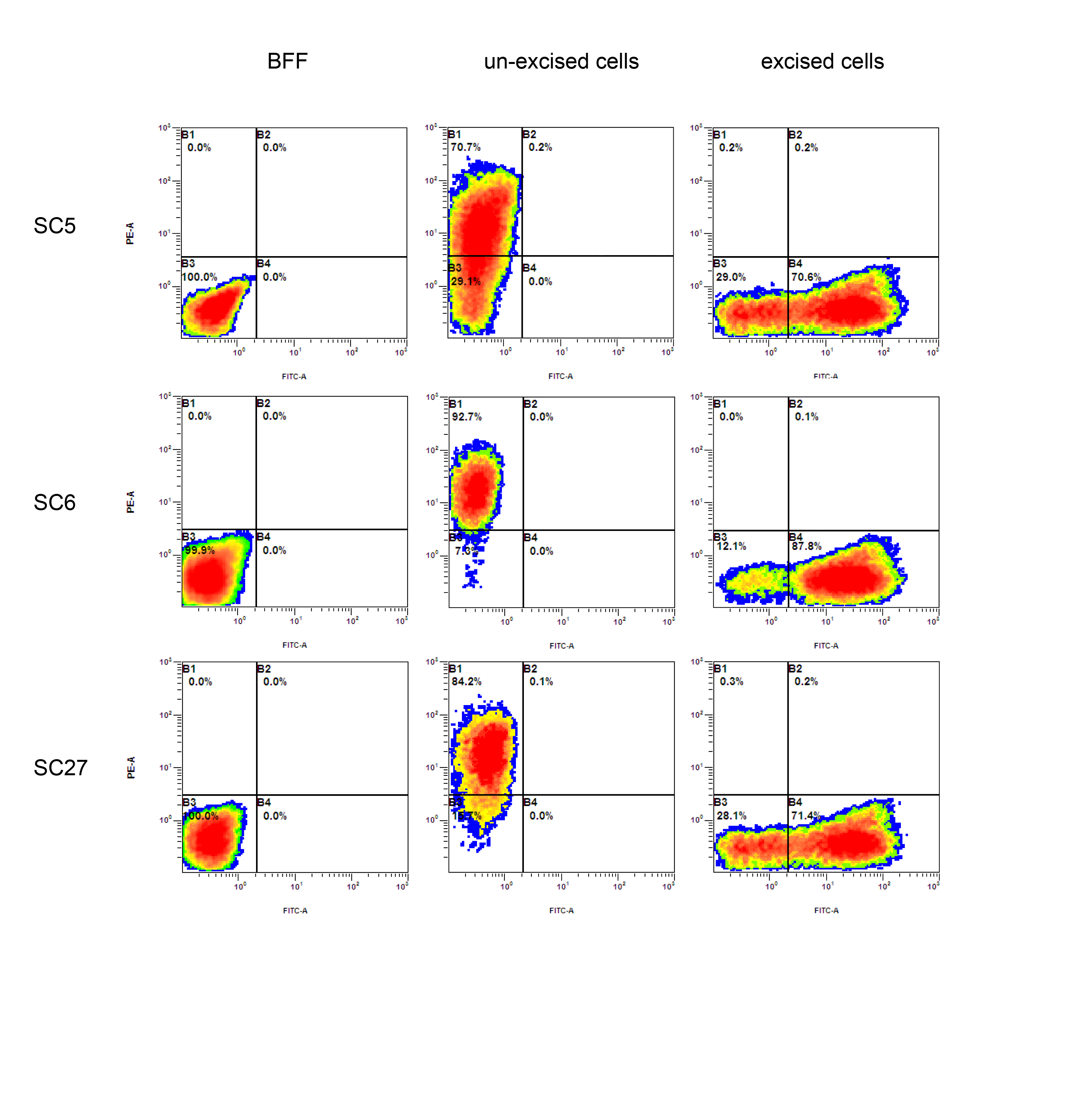

Supplement: Figure S2 — FACS analysis after His-NLS-TAT-Cre protein transduction into three RFP+ cells lines. At Day 5 after protein transduction, cells were trypsinized and resuspended in PBS containing 10% FBS, and then analyzed for RFP and GFP expression by flow cytometry. More than 70% of the transduced cells were GFP+ as shown by flow cytometry. BFF cells were untransfected control. (TIF) [file pone.0062457.s002.tif]

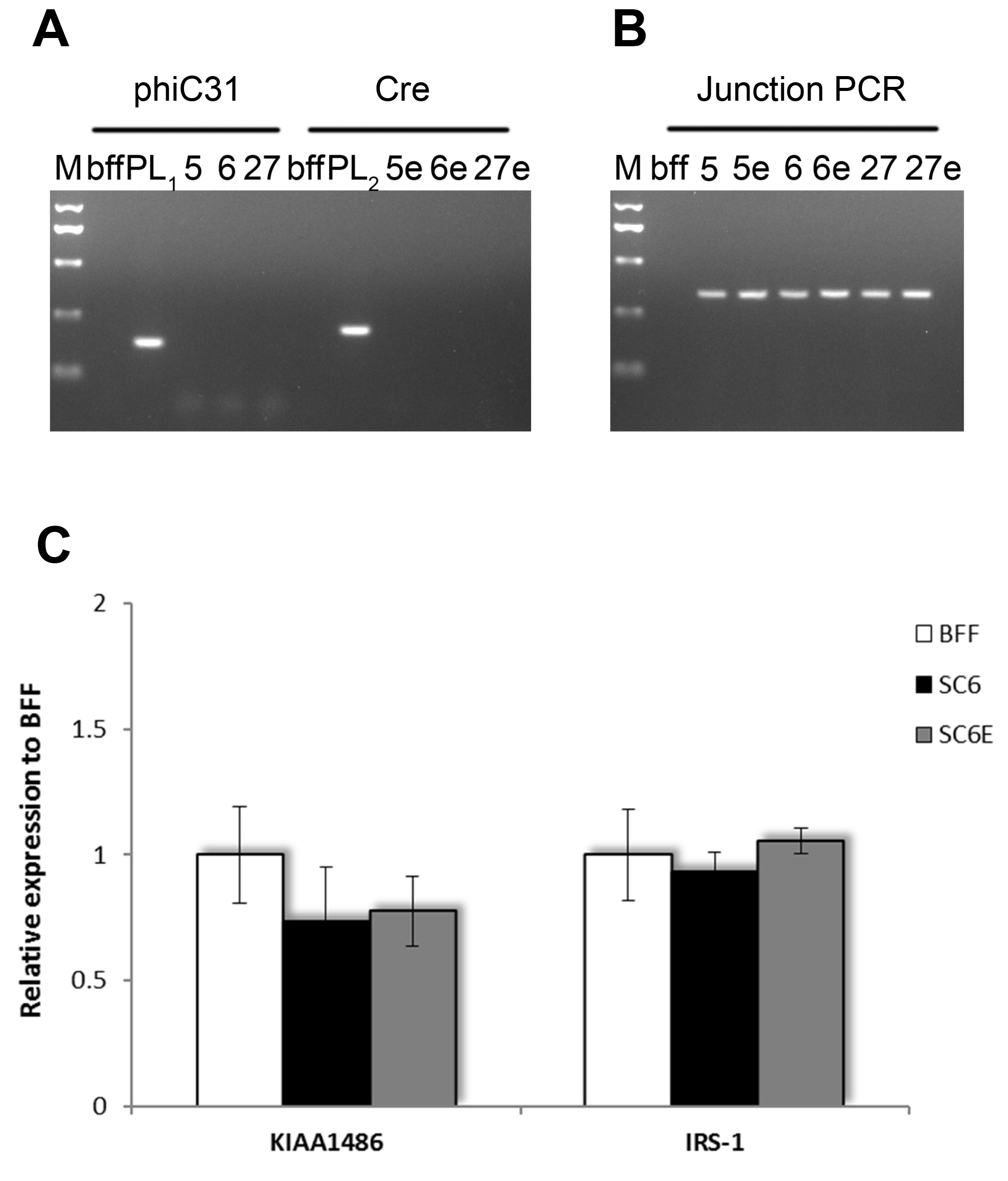

Supplement: Figure S3 — Further analysis of antibiotic selectable marker free transgenic cells. (a) PCR to test for pCMVInt and pCAG-Cre-IP showing the absence of either phiC31 integrase or Cre recombinase encoding DNA in established excised transgenic colonies. Genomic DNA from untransfected bovine fetal fibroblasts was used as a negative control, and PL1 (pCMVInt plasmid) and PL2 (pCAG-Cre-IP plasmid) were used as positive controls. (b) Verification of the genomic integration sites of the established excised cells by junction PCR using pairs of the respective genomic and plasmid-binding primers. (c) Relative real-time RT-PCR analysis of KIAA1486 and IRS-1 gene expression in un-excised and excised transgenic cells compared with that in untransfected bovine fetal fibroblasts normalized to 1. Error bars denote SEM. (TIF) [file pone.0062457.s003.tif]

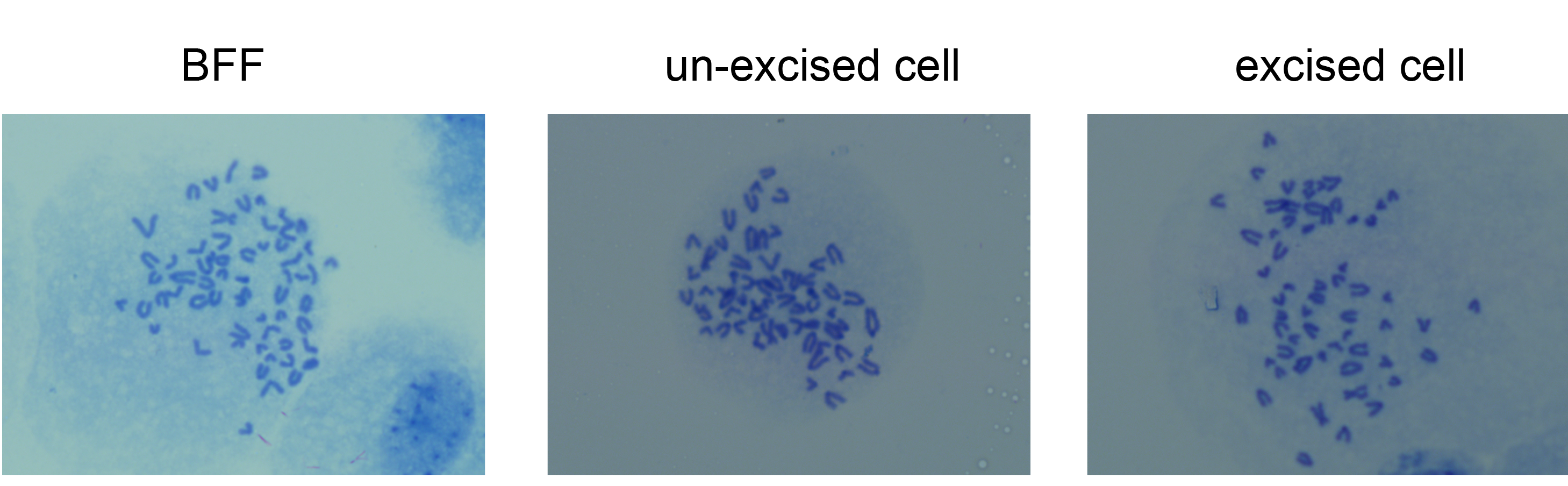

Supplement: Figure S4 — Chromosome counts. Metaphase spreads of un-excised and excised transgenic cells were counted and compared to untransfected bovine fetal fibroblasts. (TIF) [file pone.0062457.s004.tif]

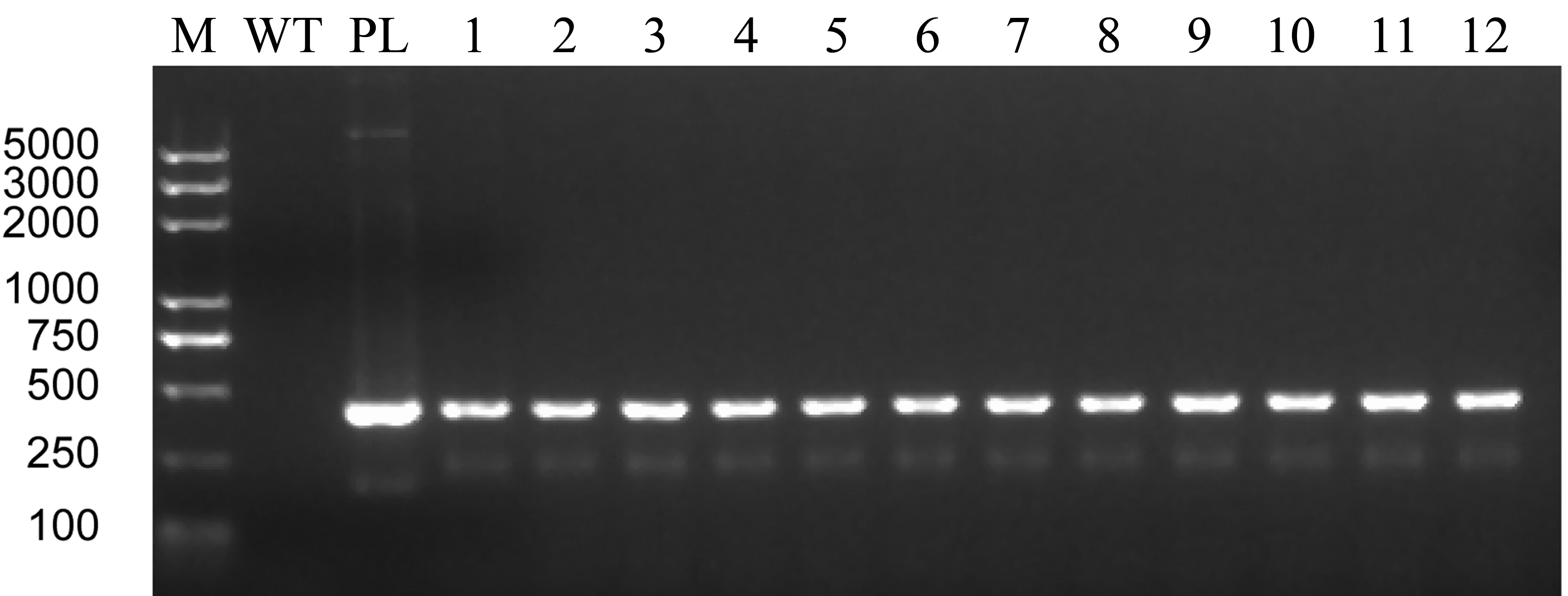

Supplement: Figure S5 — PCR analysis of transgenic cattle. M, DNA ladder; WT, non-transgenic cattle; and PL, positive control from the constructed vector; line 1–12, marker-free transgenic cattle mTG1-12. (TIF) [file pone.0062457.s005.tif]

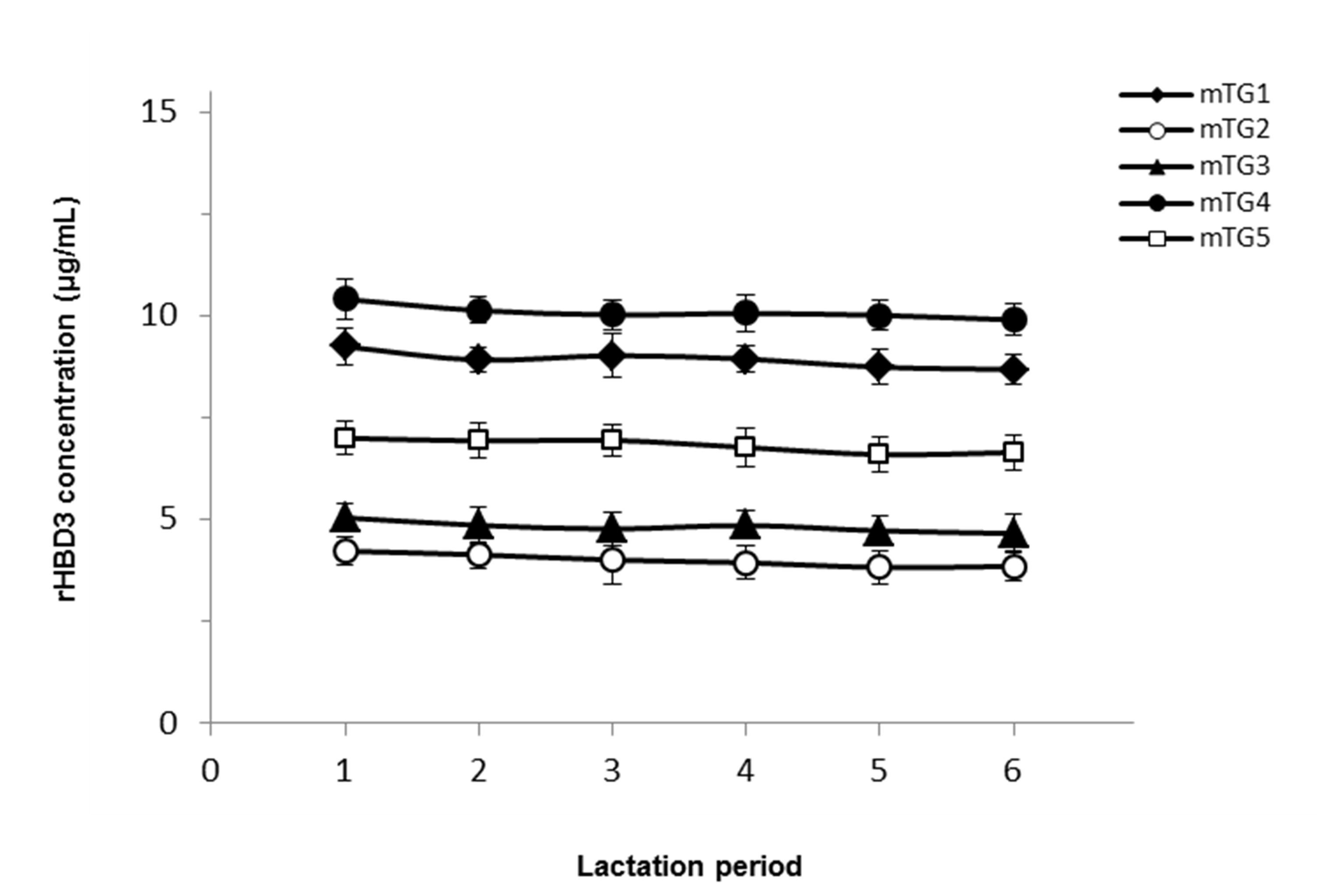

Supplement: Figure S6 — Human β-defensin-3 concentration during the first lactation period of transgenic cows. Milk was collected once a month for 6 months. Error bars denote SEM. (TIF) [file pone.0062457.s006.tif]

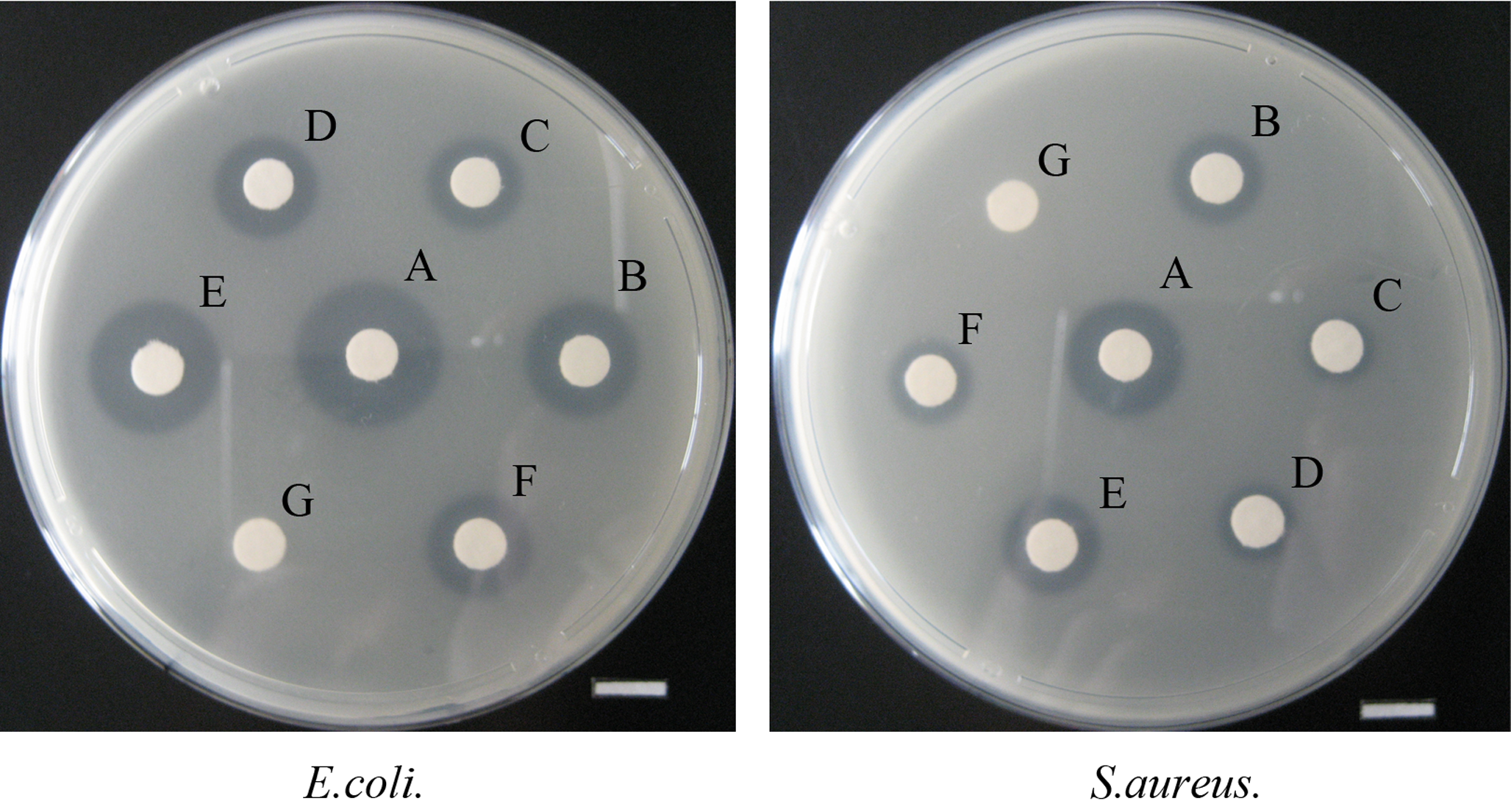

Supplement: Figure S7 — Lytic activity of human β-defensin-3 and milk samples from transgenic cows against S. aureus and E. coli . The small circles (6 mm in diameter) consist of quantitative filter paper with 10 µL of test sample or skim milk from non-transgenic cows (control). The larger circles are the inhibition zones. A, commercial human β-defensin-3; B–F, milk samples from transgenic cows mTG1–5; and G, milk samples from non-transgenic cows (control). The bar indicates 10 mm. (TIF) [file pone.0062457.s007.tif]

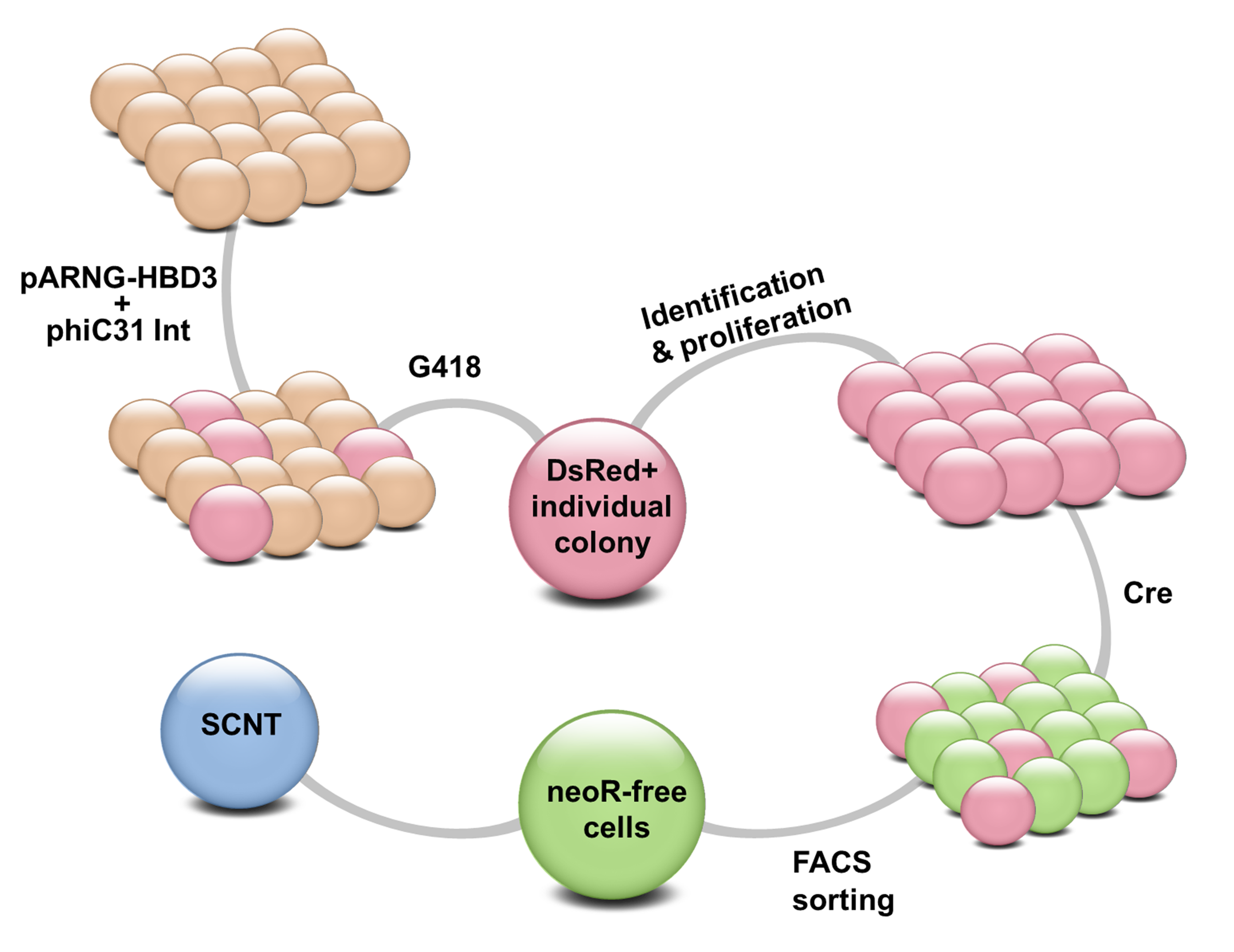

Supplement: Figure S8 — Generation of antibiotic selectable marker free transgenic cells using site-specific recombinase. To generate competent transgenic donor cells for SCNT efficiently, five main steps were used in our study as follows. Step 1: Co-electroporation with the transgene incoming plasmid and phiC31 integrase mRNA; Step 2: Generation of G418-resistant and RFP+ colonies; Step 3: Identification (attB cleavage assay → copy number analysis of colonies with cleaved attB site → integration site analysis of single-copy integrants) and proliferation; Step 4: Cre protein transduction into “safe harbor” integrated colonies; Step 5: Sorting cells showing both GFP+ and RFP− by FACS for SCNT. (TIF) [file pone.0062457.s008.tif]
