# Supplementary material for: A Site-Specific Recombinase-Based Method to Produce Antibiotic Selectable Marker Free Transgenic Cattle
Source: PLoS One. 2013 May 1;8(5):e62457. doi: 10.1371/journal.pone.0062457 (PMC3641042; doi:10.1371/journal.pone.0062457)
Supplement: Table S2 — Primers used for SOE-PCR, gene amplification, qRT-PCR and the detection of pCMVInt and pCAG-Cre-IP. (DOCX) [file pone.0062457.s010.docx]

Table S2. Primers used for SOE-PCR, gene amplification, qRT-PCR and the detection of pCMVInt and pCAG-Cre-IP

| **Primer** | **Sequence** | **Restriction site** | **Template** |
| --- | --- | --- | --- |
| attB1 F | TTCGACGCGTAGTTATTAATGTCGACGATGTAGGTCACGGTCTCGAAGCCGCGG | AseI |  |
| attB1 R | CGCGCCCGGGGAGCCCAAGGGCACGCCCTGGCACCCGCACCGCGGCTTCGAGAC |  |  |
| attB2 F | GGCTCCCCGGGCGCGTACTCCACCTCACCCATCTGGTCCATCATGATGAACGGG |  |  |
| attB2 R | GCGCCGCGCGTTCGCCGGGATCAACTACCGCCACCTCGACCCGTTCATCATGAT |  |  |
| attB3 F | GCGAACGCGCGGCGCACCGGGAAGCCCTCGCCCTCGAAACCGCTGGGCGCGGTG |  |  |
| attB3 R | ACCCGCCGACGCCGTCGCACGTCCCGTGCTCACCGTGACCACCGCGCCCAGCGG |  |  |
| attB4 F | ACGGCGTCGGCGGGTGCGGATACGCGGGGCAGCGTCAGCGGGTTCTCGACGGTC |  |  |
| attB4 R | AACGACGCGTTACTATTAATGTCGACATGCCCGCCGTGACCGTCGAGAACCCGC | AseI |  |
| LoxPMSC F1 | CTAGCTAGCTAGATAACTTCGTATAGCATACATTATACGAAGTTATTTGGCGCGCCTTG | NheI |  |
| LoxPMSC R1 | TAGTTTAGCGGCCGCAAATGCCTTAAGATGGACTAGTCCAAGGCGCGCCAAATAAC |  |  |
| LoxPMSC R2 | GCGACCGGTAGATAACTTCGTATAATGTATGCTATACGAAGTTATAGTTTAGCGGCCGC | AgeI |  |
| MCS1 F | TCGACGCGTCGCGGATCCGTGCCTTAATTAAGGACTGGGCCCATTGCGAGCTC | MluI |  |
| MCS1 R | CTCGAGGGTGCAACGTTCGCTTGGCCGGCCTTGACCGAGCTCGCAATGGGCCC |  |  |
| MCS2 F | AACGTTGCACCCTCGAGCGGATCGATCGATTGGGGTACCCCGCCAAGCTTGG |  |  |
| MCS2 R | ATGGCAGGGCCTGCCGGAATTCGGTGAAGATCTTCTCCCAAGCTTGGCGGGG | EcoO109I |  |
| Kneo F | GGACTAGTGCGTCAGGTGGCACTTTTCG | SpeI | pEGFP-C1(Clontech) |
| Kneo R | AGCCTTAAGCCCCGACGTTGGCTG | AflII | pEGFP-C1(Clontech) |
| SV40pa F | CCATCTTAAGTGATCATAATCAGCCATACCAC | AflII | pEGFP-C1(Clontech) |
| SV40pa R | TAAAGCGGCCGCTAAGATACATTGATGAG | NotI | pEGFP-C1(Clontech) |
| DsRedpA F | ATAGGCGCGCCACCATGGTGCG | AscI | pDsRed1-B2 |
| DsRedpA R | CCGGACTAGTCATTGATGAGTTTGGACAAACCAC | SpeI | pDsRed1-B2 |
| IVTInt F | CTAGCTAGCGCCACCATGACACAAGGGGTTGTGACC | NheI | pCMVInt^[^[^1^](#_ENREF_1)^]^ |
| IVTInt R | CTGACTTAAGTTAAACTTTACGTTTTTTCGGCGCCGCTACGTCTTC | AflII | pCMVInt^[^[^1^](#_ENREF_1)^]^ |
| HNI F1 | TAGCGGTTTGACTCACG |  |  |
| HNI F2 | CCCATAGTAACGCCAATA |  |  |
| HNI R | CGATGTAGGTCACGGTCT |  |  |
| qKIAA1486 F | CGACATGCCAAACGCTTTC |  |  |
| qKIAA1486 R | CACAGACGGCCACAGCTTATC |  |  |
| qIRS-1 F | CATAGACCTGGATTTGGTCAAGGA |  |  |
| qIRS-1 R | GACTACTGGCCAGAGGCTGATG |  |  |
| qGAPDH F | TCAACGGGAAGCTCACTGG |  |  |
| qGAPDH R | CCCCAGCATCGAAGGTAGA |  |  |
| qRFPstd F | GCCACAACACCGTGAAGCTGAA |  |  |
| qRFPstd R | ACACCTTGGAGCCGTACTGGAA |  |  |
| NLSTAT F | TATGCCGAAAAAAAAACGTAAAGTTTACGGTCGTAAAAAACGTCGTCAGCGTCGTCGTGAGCT | NdeI |  |
| NLSTAT R | ACGACGACGCTGACGACGTTTTTTACGACCGTAAACTTTACGTTTTTTTTTCGGCA | SacI |  |
| CreTAA F | TTCGAGCTCATGTCCAATTTACTGACCG | SacI | pCAG-Cre-IP^[^[^2^](#_ENREF_2)^]^ |
| CreTAA R | CCGCTCGAGCTAATCGCCATCTTC | XhoI | pCAG-Cre-IP^[^[^2^](#_ENREF_2)^]^ |
| BFF2 junction F | GACGATGTAGGTCACGGTCTCG |  |  |
| BFF2 junction R | GCAGCAGCAGTCACTAAGTTGT |  |  |
| ProbeGFP F | AAGTTCATCTGCACCACCG |  | pARNG-HBD3 |
| ProbeGFP R | TGCTCAGGTAGTGGTTGTCG |  | pARNG-HBD3 |
| TestC31 F | CAACCGTTATGCGAATCCTTC |  |  |
| TestC31 R | ATGATCGGTCCGCAATCAA |  |  |
| TestCre F | AATGCTTCTGTCCGTTTGC |  |  |
| TestCre R | ATTGCTGTCACTTGGTCGTG |  |  |
| TestHBD3 F | CTTCCTAAAACCTTTCCGTG |  |  |
| TestHBD3 R | CCAGTCGCAGTCAATTCTGT |  |  |

1. Groth AC, Olivares EC, Thyagarajan B, Calos MP (2000) A phage integrase directs efficient site-specific integration in human cells. Proc Natl Acad Sci U S A 97: 5995-6000.

2. Li P, Tong C, Mehrian-Shai R, Jia L, Wu N, et al. (2008) Germline competent embryonic stem cells derived from rat blastocysts. Cell 135: 1299-1310.
